# Changes in Motor Strategy and Neuromuscular Control During Balance Tasks in People with a Bimalleolar Ankle Fracture: A Preliminary and Exploratory Study

**DOI:** 10.3390/s24216798

**Published:** 2024-10-23

**Authors:** Diana Salas-Gómez, David Barbado, Pascual Sánchez-Juan, María Isabel Pérez-Núñez, Esther Laguna-Bercero, Saray Lantarón-Juarez, Mario Fernandez-Gorgojo

**Affiliations:** 1 Escuelas Universitarias Gimbernat (EUG), Physiotherapy School Cantabria, University of Cantabria, 39300 Torrelavega, Spain; diana.salas.gom@gmail.com (D.S.-G.); saray.lantaron@eug.es (S.L.-J.); 2 Sports Research Centre, Department of Sport Science, Miguel Hernández University of Elche, 03202 Elche, Spain; 3 Alicante Institute for Health and Biomedical Research (ISABIAL), 03550 Alicante, Spain; 4 Alzheimer’s Centre Reina Sofia-CIEN Foundation, 28031 Madrid, Spain; psanchezjuan@fundacioncien.es; 5 Neurodegenerative Disease Network Biomedical Research Center (CIBERNED), 28029 Madrid, Spain; 6 Traumatology Service and Orthopedic Surgery, University Hospital “Marqués de Valdecilla” (UHMV), 39008 Santander, Spain; isabel.perez@unican.es (M.I.P.-N.); mesther.laguna@scsalud.es (E.L.-B.); 7 Nursing and Physical Therapy Department, Faculty of Health Sciences, University of Leon, 24401 Ponferrada, Spain; mafeg@unileon.es

**Keywords:** electromyography, motor strategy, postural control, lower limb biomechanics, dynamic balance

## Abstract

Ankle fractures can lead to issues such as limited dorsiflexion, strength deficits, swelling, stiffness, balance disorders, and functional limitations, which complicate daily activities. This study aimed to describe neuromuscular adaptations at 6 and 12 months post-surgery during static and dynamic balance tasks, specifically using the Y-Balance Test (YBT). Additionally, the relationship between neuromuscular patterns, balance, and musculoskeletal deficits was evaluated. In 21 participants (14 at 6 months and 21 at 12 months) with bimalleolar fractures, hip strength, ankle dorsiflexion, ankle functionality, and static and dynamic balance were assessed using electromyography of five lower limb muscles (tibialis anterior, peroneus longus, lateral gastrocnemius, biceps femoris, and gluteus medius). A significant interaction effect (limb × proximal [hip]—distal [ankle] muscle) (F = 30.806, *p* < 0.001) was observed in the anterior direction of the Y-Balance Test (YBT_A_) at 6 months post-surgery. During the YBT_A_ and YBT posteromedial (YBT_PM_), it was found that a lower dorsiflexion range of movement was associated specifically at 6 months with greater activation of the lateral gastrocnemius. However, these differences tended to diminish by 12 months. These findings suggest that neuromuscular patterns differ between operated and non-operated limbs during the YBT_A_ at 6 months post-surgery. The Y-Balance Test, particularly its anterior direction, effectively highlighted these neuromuscular changes. This is a preliminary study; further research is needed to explore these findings in depth.

## 1. Introduction

Ankle fractures are among the most common traumatic injuries affecting the lower limbs, often necessitating surgical intervention when the fractures are unstable [1]. These injuries typically result in various complications throughout the affected limb, including significant limitations in dorsiflexion, deficits in strength, swelling, stiffness, and functional impairments that adversely impact daily activities [2,3,4,5]. Post-fracture balance is notably compromised, especially during challenging tasks that involve single-leg support or dynamic activities, as shown by studies using the Star Excursion Balance Test (SEBT) or the Y-Balance Test (YBT) [2,4,6,7]. Adequate postural control is contingent upon the central nervous system’s ability to integrate afferent inputs from the vestibular, visual, and somatosensory systems [8,9]. Moreover, a well-functioning neuromuscular system is critical to counterbalance the external forces generated by gravity and other disturbances, thereby maintaining postural stability and orientation relative to gravity [9,10,11]. Consequently, neuromuscular dysfunctions during balance tasks can degrade movement quality, potentially leading to prolonged functional deterioration [12]. This highlights the importance of investigating the role of lower limb musculature during balance tasks in patients who have undergone surgery for bimalleolar ankle fractures by utilizing reliable biomechanical assessment tools such as electromyography (EMG).

Although previous research on neuromuscular behavior during balance tasks in this specific group is scarce, EMG studies in different populations with ankle injuries have identified changes in muscle activation patterns. These include delayed responses in muscles such as the peroneal or tibialis anterior during various functional and/or balance-related activities [12,13,14,15]. Specifically, subjects with chronic ankle instability (CAI) show reduced activation and delayed response in the ankle muscles during transition movements from the bipodal to the unipodal position [14]. Additionally, reduced activation of the tibialis anterior muscle has been observed in functional tests such as the SEBT [12].

Furthermore, certain neuromuscular changes are not limited to the muscles involving the ankle joint but have also been detected in body regions such as in the rectus femoris, which has shown a lower amplitude of activation during balance tasks, or in the gluteus maximus of the hip, with a lower percentage of activation during the SEBT or rotational squat [12,16,17,18]. These changes in distant regions may represent adaptive or compensatory mechanisms that patients adopt or develop to execute specific functional tasks [19]. Consequently, analyzing the musculature of the lower limb through surface electromyography in balance or functional tasks, such as the YBT, could offer a deeper understanding of the neuromuscular strategy employed to preserve postural stability in individuals with bimalleolar ankle fractures. This information can be of substantial clinical importance, as it provides clinicians with valuable insights to better guide and personalize rehabilitation and enhance recovery in those with ankle fractures.

Drawing on the neuromuscular findings observed in other populations with ankle injuries and considering the characteristics of individuals who have undergone surgical treatment for bimalleolar ankle fractures, it is plausible to assume that neuromuscular alterations could occur in the operated lower limbs post-fracture, potentially contributing to balance impairments.

In this regard, a previous study by our research group examined the static balance of people with ankle fractures using a pressure platform and dynamic balance using the YBT [20]. The study revealed significant deficits in static and dynamic balance at mid-term (6 months) and dynamic balance deficits at long-term (12 months) post-surgery. In addition, at 12 months post-surgery, patients still showed a significantly reduced dorsal ankle flexion. Finally, it was found that deficits in dorsiflexion and hip strength were important factors explaining the deficits in dynamic balance at 12 months.

However, in that study, we did not present the analysis of the neuromuscular behavior of lower limb muscles shown by those persons with ankle fractures during the balance tasks. In this regard, it is also essential to know the neuromuscular control during the execution of these tests in order to identify in depth if there are any neuromuscular factors that explain these balance deficits.

The availability of information on clinical, biomechanical, and neuromuscular balance assessment could help clinicians choose specific assessment tests and rehabilitation exercises according to the deficits and goals that are set, as well as a thorough and complete follow-up during the rehabilitation process.

Therefore, the primary goal of this study was to identify and characterize the neuromuscular adjustments at mid-term (6 months) and long-term (12 months) following surgery in patients with bimalleolar ankle fractures during both static and dynamic balance exercises. Additionally, this study aimed to assess the relationship between neuromuscular patterns, balance, functionality, and the musculoskeletal deficits observed in this group.

## 2. Materials and Methods

This longitudinal study involved 21 participants who underwent bimalleolar ankle fracture surgery at the Traumatology Unit of Marqués de Valdecilla Hospital (HUMV). Inclusion criteria included: (1) surgery performed using the Open Reduction and Internal Fixation (ORIF) technique by two specific orthopedic surgeons; (2) adherence to a standardized post-surgery protocol involving immobilization, discharge, and rehabilitation; and (3) age between 18 and 55 years. Exclusion criteria encompassed the following: (1) bilateral limb involvement; (2) neurological or rheumatological conditions; (3) open or tibial pylon fractures; and (4) incomplete follow-up. Following an immobilization period of 3.4 ± 1.2 weeks, a 6-week progressive rehabilitation program was initiated. This included passive stretching, kinesitherapy, and exercises to strengthen the ankle. Upon receiving approval from the orthopedic surgical team, participants began a 3.2 ± 2.5-month program focused on balance, proprioception, and gait training. These rehabilitation sessions were conducted five days a week, each lasting about 45 min, and were overseen by the physiotherapy team at HUMV. The study adhered to the Declaration of Helsinki and received approval from the Cantabria Clinical Research Ethics Committee at Marqués de Valdecilla Hospital (reference: 2017.072).

### 2.1. Procedure

The research was carried out in a Laboratory of Movement Analysis. Two evaluation sessions were conducted at 6 and 12 months post-ankle surgery, with each session lasting 2 h. These sessions consisted of three parts: (1) descriptive and anthropometric measurements (such as body mass and height), (2) completion of the American Orthopedic Foot & Ankle Society (AOFAS) questionnaire, and (3) assessment of hip strength, ankle dorsiflexion range of motion (ROM), and static and dynamic balance with EMG surface evaluation. The order of measurement and tests was the same for all participants.

#### 2.1.1. Descriptive and Anthropometric Measurements

The following information was reported for each participant: age (years), sex (male or female), height (cm), and weight (kg).

#### 2.1.2. Ankle Functionality Questionnaire Post-Surgery

The American Orthopedic Foot & Ankle Society (AOFAS) scale was utilized to evaluate ankle functionality following surgery. The AOFAS provides a score out of a maximum of 100 points, with a higher score denoting better functionality. It is divided into three categories: pain (up to 40 points), functionality (up to 50 points), and alignment (up to 10 points) [21].

#### 2.1.3. Hip Strength and Ankle Range of Movement

Maximal hip abduction force during an isometric contraction was measured using a hand-held dynamometer (microFET@2, Hoggan Scientific L.L.C, Salt Lake City, UT, USA) [22]. Participants lay supine on a stretcher with knees extended and arms parallel to the body. The dynamometer was placed in contact with the outside of the knee. They performed a warm-up consisting of two progressive trials before the test. Subsequently, they completed three trials with a one-minute rest between each. Participants were instructed to progressively reach their maximum strength within a 5 s window and were verbally encouraged during each trial. This method has demonstrated very high reliability (ICC > 0.9) [7]. The strength of the abductors of both hips was assessed.

On the other hand, the evaluation of ankle dorsiflexion ROM was specifically assessed using the weight-bearing lunge (WBL) method with the knee flexed, as previously described [23]. For this purpose, a digital inclinometer was placed on the tibial tuberosity for reliable measurement (Acumar, Lafayette Instrument, Lafayette, IN, USA). This method has demonstrated very high reliability (ICC > 0.9) [24]. Each limb was tested three times to allow the tissues to adapt to the position. Participants stood barefoot, facing a wall, with the foot to be tested positioned 30 cm from the wall, keeping the arms in contact with the wall and the knee aligned with the second toe. From this position, participants bent their knee towards the wall until they reached their maximum ankle dorsiflexion ROM [25,26].

#### 2.1.4. Instrumentation and Electrode Placement for Muscle Activity

Evaluation Muscle activity was recorded using a 16-channel wireless electromyography system (DTS DESKTOP myoMUSCLE^®^, Noraxon, 15770 North Greenway-Hayden Loop, Suite 100, Scottsdale, AZ 85260, USA) [27]. Each sensor consists of a signal preamplifier (1st-order high-pass filters set to 10 Hz +/− 10% cutoff, baseline noise < 1 uV RMS, input impedance > 100 Mohm, input range/−6.3 mV, CMR 100 dB, base gain 200, and final gain 500). Two clamps are connected to this preamplifier, and they are attached to the electrodes placed on the muscles to be recorded. The preamplifier sends the signal to a receiver, which is connected to the computer via USB. The EMG system was operated using NORAXON software 3.6 [28]. The recording was made at a sampling rate of 1500 Hz.

For the placement of the sensors, the SENIAM recommendations were followed [29]. The muscle groups recorded bilaterally were as follows: anterior tibialis (a mark was made at 1/3 of the line between the most prominent area of the fibula and the most prominent area of the medial malleolus), lateral peroneal (a mark was made at 25% of the line between the most prominent area of the fibula head and the most prominent area of the lateral malleolus), lateral gastrocnemius (a mark was made at 1/3 of the line between the fibula head and the heel), biceps femoris (a mark was made at 50% on the line between the ischial tuberosity and the lateral epicondyle of the tibia), and gluteus medius (a mark was made at 50% of the distance between the iliac crest and the greater trochanter). Subsequently, the skin was prepared. First, the skin area was shaved and then cleaned with cotton moistened with alcohol. The skin was allowed to dry, and then MIOTRACE foam electrodes with conductive hydrogel adhesive from Covidiem, 38 mm in size, were placed. The distance between electrodes was 20 mm, and they were positioned parallel to the muscle fibers. After this, the EMG sensors were placed, and the amplifiers were adhered to the skin using double-sided adhesive tape and positioned in an area of the skin where they would not interfere with the balance tests. To normalize the EMG signal, 2 maximal voluntary isometric contractions (MVIC) of each muscle group were performed [30]. The maximum effort was maintained for 5 s to reach the peak maximum effort. For the anterior tibialis and lateral gastrocnemius, a dorsiflexion and a plantar flexion were performed in a 90° flexion position of the ankle, respectively. For the lateral peroneal, an eversion was performed in a 45° flexion position of the ankle. For the biceps femoris, a knee flexion was performed in a 45° flexion position [9]. Finally, for the gluteus medius, a hip abduction was performed from the neutral position of the hip. Participants were verbally encouraged during the test. To avoid fatigue, the limbs were alternated, respecting a rest period for the same muscle group of 2 min.

#### 2.1.5. Balance Assessment

After the placement of the EMG and subsequent tests for its normalization, static balance and dynamic balance were evaluated using the YBT (Y-Balance Test Kit™, Move2Perform, Evansville, IN, USA) [31], a highly reliable tool for assessing dynamic balance in people with ankle injuries [7,31].

##### Static Balance

Static balance was assessed through single-leg and tandem stance tests. Specifically, participants performed five balance tests in the following order: (1) single-leg stance with the non-operated limb and open eyes, (2) single-leg stance with the operated limb and open eyes, (3) single-leg stance with the non-operated limb and closed eyes, (4) single-leg stance with the operated limb and closed eyes, and (5) tandem position with the operated limb behind the non-operated limb and open eyes. Balance trials were alternated between the non-operated and operated limbs to prevent fatigue. Before testing, participants had a brief familiarization period. The recording duration for each test was 30 s, with a 30 s rest in between. Each task was repeated twice.

##### Dynamic Balance

Participants’ dynamic balance was assessed with the Y-Balance Test, which consisted of reaching balance tasks in three directions (anterior, posteromedial, and posterolateral) with both limbs. The Y-balance kit uses a central plastic plate for the supporting barefoot (tested limb), with three tubes located in the anterior, posteromedial, and posterolateral directions, each with a mobile plastic box. While standing with a single leg on the central box, participants were instructed to ‘reach as far as possible by moving the mobile box with the free foot and then return without the plate to the starting position without losing balance’. A brief familiarization period of two trials in each direction was conducted. This was followed by test trials with a 20 s rest between each trial. Participants could make several attempts until two valid trials were completed. Participants were allowed to spend as much time as they deemed necessary to complete each trial. Typically, participants spent between 2 and 6 s to complete each trial. Operated and non-operated limb trials were alternated to avoid fatigue. A trial was considered unsuccessful if the non-tested limb touched the floor, the participant placed their foot on top of the box, or if they lost contact with the box during the reach [20].

### 2.2. Data Reduction

For hip abduction, the highest peak of force obtained in any trial was selected and normalized to each participant’s body mass (kg*100/body mass) for the subsequent statistical analyses. For the ankle ROM, the test was performed three times, and the two most similar values were averaged [23].

The EMG signal obtained in all tasks was filtered with a band-pass filter of 15–450 Hz (Butterworth, 4th order). Subsequently, the signal was rectified and smoothed using a 20 ms moving average filter. A 20 ms smoothing window was selected because short time windows are used for the electromyographic analysis of rapid or short-duration movements such as those that occur in balance tasks, which are controlled by bursts of intermittent nerve impulses to produce rapid postural adjustments [8,9,10,32]. Finally, the EMG signal was normalized based on maximum voluntary isometric contractions (MVICs) or maximum voluntary contractions (MVCs) for each muscle.

In the static and dynamic balance tests, the EMG data from the trials were averaged. In static balance tasks, the normalized mean amplitude was determined during the middle 20 s of the task, as the first 5 s and last 5 s of each trial were discarded to reduce nonstationary signals [33]. Considering that the execution time was not standardized for the dynamic balance tasks, but rather each subject used the time needed to perform the test that ranged between 2 and 6 s, the maximum average of one-second EMG moving windows was calculated for each y-balance task. Specifically, a new time series was calculated from the rectified and normalized EMG signal, in which each new data point was computed as the average EMG activity of a one-second moving window. Then, the maximum value was used for the subsequent statistical analyses.

To simplify the EMG analysis and gain a more comprehensive insight into the EMG activity across the entire lower limbs during balance tasks, the cluster muscle activity was calculated for both the distal muscles at the ankle joint and the proximal muscles at the hip joint in addition to the individual calculations for each muscle [17]. For this, the data from the anterior tibialis, long peroneal, and lateral gastrocnemius were averaged for the distal musculature and the biceps femoris and gluteus medius for the proximal musculature.

### 2.3. Statistical Analysis

The normality of the distribution of variables was checked using the Shapiro–Wilk test. Descriptive statistics for all variables (mean and standard deviation) were calculated. To investigate the differences between the operated and non-operated limb in muscle activation of each muscle, two-way repeated measures analyses of variance (ANOVAs) were conducted, with the limb category (2 levels: operated or non-operated) and muscle category (5 levels: anterior tibialis, long peroneal, lateral gastrocnemius, biceps femoris, or gluteus medius) as within-subject factors. Likewise, repeated measures ANOVAs were performed to assess differences in the activation of distal and proximal musculature in each of the limbs or between limbs, with the limb category (2 levels: operated or non-operated) and joint category (2 levels: distal (ankle) or proximal(hip)) as within-subject factors. Pairwise comparisons were made using t-tests and corrected with the Bonferroni adjustment. Hedges’ g index (g) for paired data and its 95% confidence interval were used as estimators of effect size. A comparison was considered statistically significant when the confidence interval of the effect size did not cross the zero value. Furthermore, the practical significance of the effect sizes was interpreted as trivial (g < 0.2), small (0.2 ≤ g < 0.5), moderate (0.5 ≤ g < 0.8), and large (g ≥ 0.8) [34]. For Hedges’ g, positive or negative scores indicate greater or lesser muscle activation of the operated limb compared to the non-operated limb. Finally, a Pearson correlation analysis with Bonferroni adjustment was performed to evaluate the relationship between neuromuscular parameters, balance, functionality, age, and physical capacities, which showed significant differences between limbs. The statistical package SPSS (IBM SPSS Statistical v.27) was used for the analysis with a significance level of *p* < 0.05.

## 3. Results

Of the 21 participants, only 14 could be assessed with EMG at 6 months and all (21) at 12 months after the intervention (Table 1). Only 14 participants could be assessed at 6 months due to difficulties in performing the MVCs. Specifically in the ankle muscle asessments, these subjects had pain, difficulty, or fear in performing test contractions.

### 3.1. Muscle Activity During Static Balance Tests

In general, the participants with bimalleolar ankle fractures experienced neuromuscular impairments in the operated limb at 6 months, mainly in dynamic balance. However, at 12 months, these differences tended to diminish. The Y-Balance Test, in particular its anterior direction, effectively highlights these neuromuscular alterations. The specific results are shown below.

**Figure 1:**
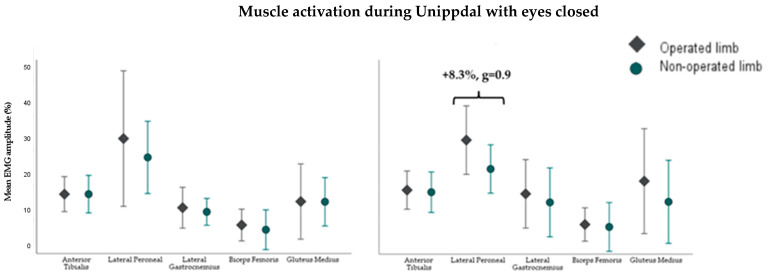
Muscle activity of the 5 muscles of the operated and non-operated limbs during single-leg stabilometry with the eyes-closed test at 6 (**left**) and 12 (**right**) months in 14 and 21 participants after surgery, respectively. Error bars show +/− 1 standard deviation. Mean EMG amplitude (%): average activation amplitude relative to maximum voluntary contraction (units: %).

**Figure 2:**
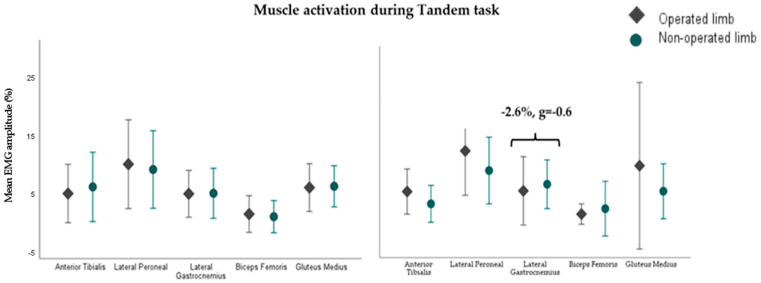
Muscle activity of the 5 muscles in the operated and the non-operated limbs during the stabilometry tandem eyes-open test at 6 (**left**) and 12 (**right**) months in 14 and 21 participants after surgery, respectively. Error bars show +/− 1 standard deviation. Mean EMG amplitude (%): average activation amplitude relative to maximum voluntary contraction (units: %).

At 6 months post-surgery, during the three static balance tests, a lower coefficient of variation was detected in the operated limb’s anterior tibialis muscle (−15%, g = −0.7) and a higher coefficient of variation in the gluteus medius (+6.4%, g = 0.6) during the single-leg support test with eyes open with respect to the non-operated limb (Figure 1). At 12 months, the operated limb in the single-leg support test with eyes closed had a higher mean activation and a lower coefficient of variation of the peroneal longus (+8.3%, g = 0.9 and −10.9%, g = −0.6, respectively) (Figure 1) and in the tandem task, a lower mean activation in the lateral gastrocnemius (−2.6%, g = −0.6) (Figure 2). Readers who wish to explore the results further can access Supplementary Material Tables S1 and S2.

**Figure 3:**
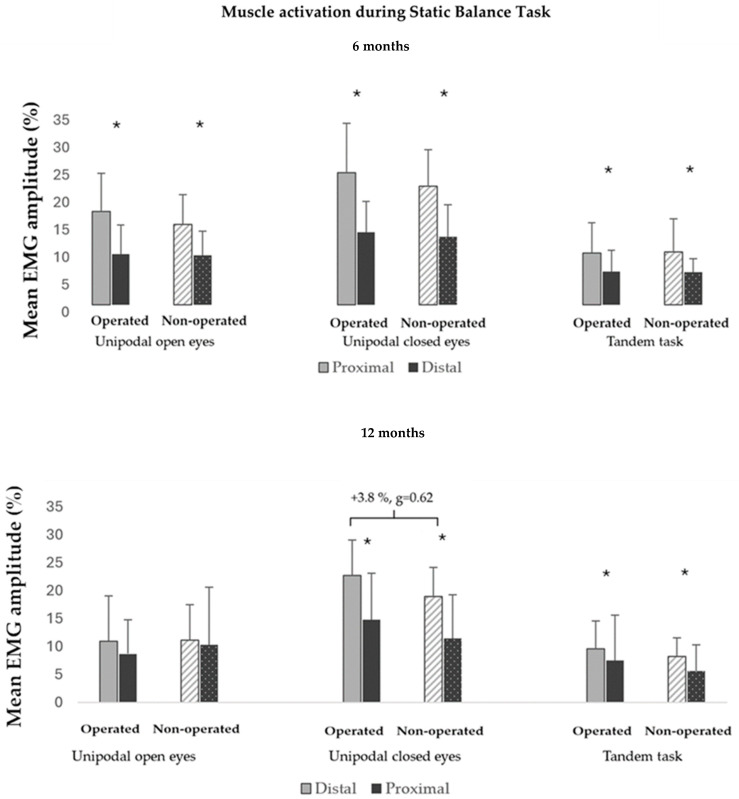
Muscle activation (distal vs. proximal) during static balance tests at 6 (**up**) and 12 (**down**) months in 14 and 21 participants after surgery, respectively. * Differences between distal and proximal muscle activation of the limb itself; } greater activation of proximal muscles during single-leg eyes-closed in the operated limb with respect to non-operated limb (+3.8%, g = 0.62) at 12 months.% MVC: average activation amplitude relative to maximum voluntary contraction (units: %).

In the analysis of muscle activation clusters, it was observed that at 6 months after surgery, the activation of the distal musculature (i.e., ankle) was greater in both lower limbs in all tasks (e.g., operated limb: +3% < mean difference > +9.4%) (Figure 3). At 12 months, there was also greater distal muscle (i.e., ankle) activation in both lower limbs in all single-leg eyes-closed and tandem tasks. In addition, greater mean ankle joint muscle activation was detected in the operated limb compared to the non-operated limb in the single-leg standing balance test with eyes closed (+3.8%, g = 0.62) (Figure 3). Readers who wish to explore the results further can access Supplementary Material Tables S3 and S4.

### 3.2. Muscle Activity During the Y-Balance Test (YBT)

**Figure 4:**
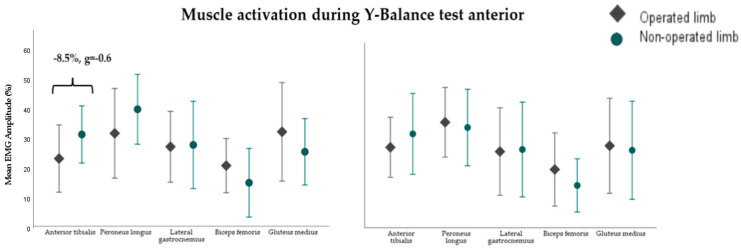
Muscle activation during the Y-Balance Test in the anterior direction of the 5 muscles of the operated and non-operated lower extremity at 6 (**left**) and 12 (**right**) months in 14 and 21 participants after surgery, respectively. %MVC: average activation amplitude relative to maximum voluntary contraction (units: %).

**Figure 5:**
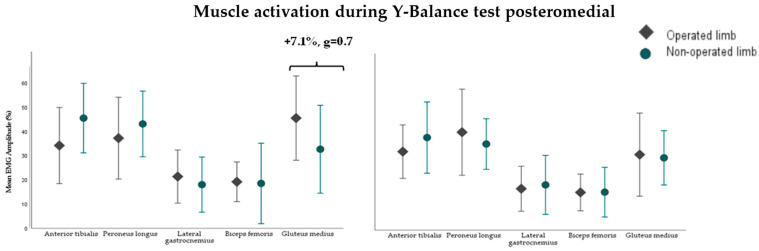
Muscle activation during the Y-Balance Test in the posteromedial direction of the 5 muscles of the operated and non-operated lower limbs at 6 (**left**) and 12 (**right**) months in 14 and 21 participants after surgery, respectively. %MVC: average activation amplitude relative to maximum voluntary contraction (units: %).

At 6 months post-surgery, the operated limb exhibited lower mean activation in the tibialis anterior muscle during the Y-Balance Test Anterior (YBT_A_) (−8.5%, g = −0.6) (Figure 4) and higher activation in the gluteus medius during the Y-Balance Test Posteromedial (YBT_PM_) (+7.1%, g = 0.7) compared to the non-operated limb (Figure 5). However, at 12 months, no significant differences in muscle activation were observed between the limbs in both YBT_A_ and YBT_PM_. Additionally, no differences in muscle activation were noted during the YBT in the posterolateral direction for the five muscles of the operated and non-operated lower limbs at both 6 and 12 months post-surgery. Readers who wish to explore the results further can access Supplementary Material Tables S5 and S6.

**Figure 6:**
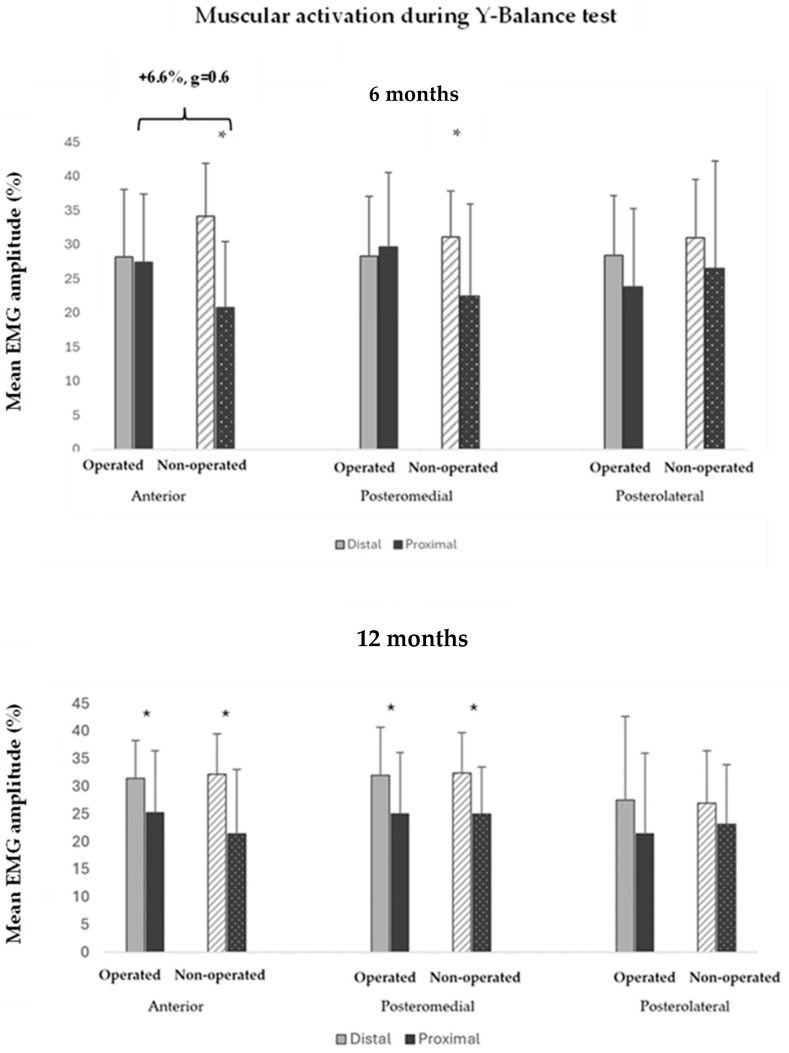
Muscle activation during the Y-Balance Test (distal vs. proximal) at 6 (**up**) and 12 (**down**) months in 14 and 21 participants after surgery, respectively. At 6 months: Y-Balance Test in the anterior direction: ANOVA interaction effect (F = 30.806 (*p* < 0.001)); Y-Balance Test in the posteromedial direction: ANOVA interaction effect (F = 4.121 (*p* = 0.065)); * differences between distal and proximal muscle activation; } greater activation of proximal muscles during YBTA in the operated limb with respect to the non-operated limb (+6.6%; g = 0.62). At 12 months, no interaction effect was found.

The ANOVA analysis of the muscle activation clusters at 6 months showed a significant interaction effect (i.e., limb proximal–distal musculature interaction) in the YBT_A_ (F = 30.806, *p* < 0.001), and in the YBT_PM_, the same trend was observed, with the interaction effect being nearly significant (F = 4.121, *p* = 0.065). This revealed that the neuromuscular pattern was different in the operated and non-operated limbs (Figure 6).

Pairwise comparisons at 6 months showed greater activation of the proximal musculature during the YBT_A_ in the operated limb compared to the non-operated limb (+6.6%, g = 0.6) (Figure 6). Regarding the comparison between musculature (distal vs. proximal) in each of the limbs, at 6 months, greater activation of the distal musculature was detected in the non-operated limb during the YBT_A_ (+13.3%, g = 1.4) and the YBT_PM_ (+8.7%, g = 0.7), while in the operated limb, the activation was similar between the distal and proximal musculature (Figure 6). At 12 months, the interaction effect (i.e., limb proximal–distal musculature interaction) in the YBT_A_ was nearly significant (F = 3.245, *p* = 0.088). The pairwise comparison showed that the distal activation compared to the proximal was greater in both lower limbs in both the YBT_A_ (operated limb: +6.2%, g = 0.6; non-operated: +10.8%, g = 1.0) and the YBT_PM_ (operated limb: +7.0%, g = 0.7; non-operated: +7.4%, g = 0.9) (Figure 6). Analysis was performed on the sample of 14 subjects at 12 months, and the results were similar. Readers who wish to explore the results further can access Supplementary Material Tables S7 and S8.

### 3.3. Relationship Between Balance, Dorsiflexion, Hip Strength, AOFAS, and Activation of Distal and Proximal Musculature

Table 2 shows the correlation results between YBT, dorsiflexion range of motion, abductor strength, ankle functionality, and muscle activity of the ankle and hip joint muscles of the operated limb at 6 and 12 months post-surgery. After the Bonferroni adjustment in the balance results, no correlation between the distance achieved in the YBT and neuromuscular patterns was found (Table 2). Concerning the FDT_ROM_ and neuromuscular patterns during the YBT, it was found that a lower FDT_ROM_ was associated specifically at 6 months with greater activation of the lateral gastrocnemius during the YBT_A_ and YBT_PM_ (−0.734 ≤ r ≤ −0.782, *p* < 0.001). In the non-operated limb, no significant correlations were detected, either with the performance of the YBT, the FDT_ROM_, or the hip ABD strength (Table 2). In addition, no relationship was observed with the stabilometry tests (Table S9). No significant correlation was found between age and neuromuscular parameters (−0.036 ≤ r ≤ 0.436, *p* > 0.05). Readers who would like to read more about the results of the static (pressure platform) and dynamic (YBT) balance assessments can find them in the previous study by Salas-Gomez et al. [20]. They can also access Supplementary Material Table S10a,b, which shows the results of the balance assessments in the 14 patients at 6 months.

## 4. Discussion

The study aimed to identify and characterize the neuromuscular adjustments at mid-term (6 months) and long-term (12 months) following surgery in patients with bimalleolar ankle fractures during both static and dynamic balance exercises. Additionally, this study aimed to assess the relationship between neuromuscular patterns, balance, functionality, and the musculoskeletal deficits observed in this group [20].

Although previous studies have identified that individuals with ankle injuries exhibit altered motor control patterns during various functional tasks [14,35], to the authors’ knowledge, this is the first work that analyzes neuromuscular behavior in people who have suffered a bimalleolar ankle fracture during balance tasks.

### 4.1. Main Results

Our findings indicate that post-surgery, patients with bimalleolar ankle fractures experience neuromuscular alterations in the operated limb at 6 months. However, by 12 months, these differences tend to diminish. The Y-Balance Test, particularly its anterior direction, effectively highlights these neuromuscular changes, supporting the notion that dynamic assessments are superior to static ones for evaluating motor control [36].

### 4.2. Neuromuscular Control in Static Balance

Based on the results, there does not appear to be a clearly altered pattern in the operated limb during static balance tests in our sample. It is worth mentioning that the differences in muscle activation in certain isolated muscles, such as the anterior tibialis or the long peroneal, tend towards greater activation in the operated limb. Only in the task of single-leg support with eyes closed was the activity of the distal musculature greater in the operated limb at 12 months post-operation. This contrasts with findings in subjects with Chronic Ankle Instability (CAI), where in this task, a lower activation of the anterior tibialis and biceps femoris was detected [17]. Concerning these discrepancies, it is essential to emphasize that comparing studies is challenging due to variances in measurement protocols. Furthermore, this study contrasts with the non-operated limb, whereas Feger et al.‘s research compares against healthy people [17]. Moreover, as previously reported in the literature, different types of ankle injuries can develop different motor strategies for balance [37,38,39]. On the other hand, and which could explain our finding, numerous authors have reported how balance tasks of greater difficulty increase muscle activation [40,41,42]. This finding leads us to believe that this task posed a greater challenge in static balance in the operated limb for our participants.

In relation to the analysis of the muscle activation cluster, in line with our results, Feger et al. found no differences in proximal or distal muscle activation in single-leg balance between subjects with CAI and healthy subjects one year after the last sprain [17]. Therefore, the fact that in our sample, no general differences are observed in proximal or distal activation between limbs and that there is a greater normalized mean amplitude of the distal musculature (i.e., ankle) compared to the proximal (i.e., hip) in both limbs suggests that these patients primarily used an ankle strategy to perform the static balance tasks. Thus, it seems that the ankle musculature of the operated limb in this sample of patients was competent to counteract the disturbances generated by this type of task [10,12,43].

### 4.3. Neuromuscular Control in Dynamic Balance

The main finding of this study was observed when analyzing dynamic reach tasks during the YBT. The interaction effect observed in the ANOVA at 6 months post-surgery suggests that the neuromuscular strategy by segments [distal (ankle) vs. proximal (hip)] was different between the operated and non-operated limb. While the non-operated limb had a dominant distal activation showing significant differences, the operated limb showed similar participation between proximal and distal musculature. Additionally, the activation of the proximal musculature was greater in the operated limb compared to the non-operated limb. Based on these findings, it appears that individuals in our sample who have undergone bimalleolar ankle fracture surgery adopt adaptive neuromuscular strategies. These strategies involve greater reliance on the proximal musculature (hip) to execute the reaching task, likely as a compensatory mechanism for musculoskeletal impairments in the distal region (ankle) [44,45], such as limited ankle dorsiflexion [2,20,46]. Also, the data indicates that patients tend to rely more on their hip muscles for the YBT_PM_ at 6 months after surgery. Despite a noticeable trend, the results were not statistically significant, likely due to the lower number of participants completing this task compared to the YBT_A_. Supporting these findings, prior research has indicated that a change in one joint may lead to modified neural activity and compensatory recruitment of muscles in adjacent joint complexes, resulting in changed movement patterns [44,47]. In line with our findings, adaptations in the proximal region have been observed in individuals with injuries to the ankle or knee [12,47,48].

Furthermore, our findings are in partial agreement with those of Gribble et al., who found that individuals with CAI exhibited increased reliance on proximal joints when performing the SEBT. This was indicated by the greater peak hip flexion required by these patients during dynamic balance tasks, as opposed to healthy controls [49]. However, these authors did not record EMG signals in the lower limb musculature, which limited their ability to fully detail the neuromuscular control strategies used by these subjects [39]. At 12 months, the neuromuscular pattern seems similar between limbs during the YBT_A_, as well as in the rest of the test directions. Our results partly coincide with those reported by Feger et al., who found no differences between CAI and controls in proximal or distal activation levels during the YBT [17]. However, it should be noted that their study did not compare the neuromuscular behaviors of the proximal and distal segments of the lower limb within each group. Consequently, they did not determine which segment contributes more significantly to performance in each direction of the test, as has been done in this study. These findings suggest the importance of the neuromuscular complex of the hip in the operated limb of individuals with bimalleolar ankle fractures to execute certain dynamic balance tasks [7].

Regarding the differences between the operated and non-operated limb in isolated muscles, we detected that even after 6 months post-surgery, the anterior tibialis muscle of the operated limb showed a lower level of activation in the YBT_A_. This coincides with what has been reported in subjects with CAI compared to healthy subjects in the SEBT [12]. However, at 12 months, the neuromuscular behavior, in general, was similar in both lower limbs. In this regard, Pozzi et al. have reported increased activation in the YBT_PM_ in the tibialis anterior and peroneus muscles in coper subjects (individuals with a history of ankle sprains but who have not developed CAI compared to healthy controls). However, no differences were observed between copers and other subjects with ankle injuries [39].

Therefore, it seems necessary to be able to compare the neuromuscular behavior of individuals with bimalleolar ankle fractures with a control group of healthy subjects since the mechanisms of the central nervous system could play a role in the functional deficits in people with bimalleolar ankle fractures, causing deficits to present bilaterally, as occurs in other ankle injuries [12,17,50].

### 4.4. Neuromuscular Behavior and Its Relationship to Balance and Musculoskeletal Parameters

In the present study, we found no correlation between performance in balance tasks and neuromuscular activation. However, Jaber et al. [12] suggested that deficits in YBT (Y-Balance Test) performance might be linked to lower neuromuscular activity. Despite patients in our study appearing to developing different strategies in their operated limb during the YBT, these strategies did not fully compensate for the previously reported deficits in our previous study [20].

A negative correlation was found between the YBT_A_ and the YBT_PM_ and distal activation; specifically, there was greater activation of the lateral gastrocnemius in those subjects with a lower dorsiflexion range of motion (FDT_ROM_). The literature reports how a limitation of ankle dorsiflexion in weight-bearing and reach tasks, such as the YBT, limits movement in the sagittal plane of the lower limb, generating compensatory movements in the frontal and transverse planes of the ankle, knee, and hip [51]. In this sense, these tasks could have involved a higher level of imbalance, which would explain the association between a lower FDT_ROM_ and a greater recruitment of lateral gastrocnemius to try to maintain stability during the YBT [52]. Finally, as reported by other authors, the differences in motor patterns between static and dynamic tasks suggest that the alterations in neuromuscular control detected in this study are task-dependent, similar to those observed in patients with CAI [17,18].

### 4.5. Limitations, Practical Implications, and Future Lines

This study has several limitations. First, although the sample size is sufficient for biomechanical research, it is not large enough to establish normative data. Second, the lack of prior studies on this specific population made it challenging to determine an optimal sample size. In relation to this, another limitation could be the high number of tasks performed and muscles that were analyzed, coupled with the limited sample size analysis. This may have influenced the results and restricted the study’s statistical power to detect significant differences or correlations between the variables analyzed. Third, the absence of a healthy control group limits our ability to benchmark the observed neuromuscular patterns against normal physiological responses. Another limitation was that the level of physical activity and other physical capacities, such as ankle strength or the strength of other limb or trunk muscles, which can influence functional recovery, were not assessed. Additionally, the Y-Balance Test was not standardized in terms of execution time or the division of the test into concentric or eccentric phases, which would have allowed for a more in-depth EMG analysis. Lastly, the use of maximum voluntary contractions (MVCs) for EMG normalization, while standard [30], may have limited the representativeness of the 6-month post-surgery assessments.

From a clinical point of view, it is important to identify and quantify objectively and early not only the quantity of movement but also the quality of the movement to make a specific therapeutic approach. Although these altered movement patterns may help patients perform certain activities and be functional to some extent, they may be less optimal or efficient motor patterns, which could lead to future injuries or overloads [37,49].

In addition, this could help with choosing specific rehabilitation exercises according to the deficits and the established goals. Based on these results, it appears that the YBT, especially in the anterior direction, is a sensitive test for detecting changes in motor strategy in subjects with bimalleolar ankle fractures. However, this is a preliminary study, and we believe that future studies are needed to further investigate this field with a larger sample size. Likewise, it would be important to evaluate neuromuscular behavior during other functional tasks and analyze whether the limitation of ankle mobility generates neuromuscular compensations in other functional tasks, such as walking, ascending, or descending stairs, similar to those observed in the YBT or others.

## 5. Conclusions

This study examined patients with bimalleolar ankle fractures at 6 and 12 months after surgery. During dynamic exercises, particularly the YBT_A_ (Y-Balance Test Anterior), neuromuscular alterations were observed in the operated limb. These alterations seemed to result from a motor strategy that involved the increased recruitment of the proximal (hip) musculature to perform the YBT_A_. A similar trend was seen in the YBT_PM_ (Y-Balance Test Posteromedial), although the interaction effect was nearly significant. However, no distinct neuromuscular pattern between limbs was found during static balance tasks. This suggests that neuromuscular adaptations or changes in motor strategy are task-dependent. Furthermore, limitations in FDT_ROM_ (dorsiflexion range of motion) led to greater recruitment of the distal (ankle) musculature in reaching tasks like the YBT (Y-Balance Test) that require extensive FDT_ROM_. One year after surgery, the motor pattern bilaterally appeared similar in both dynamic and static tasks.

## Figures and Tables

**Table 1:** Baseline characteristics of people with bimalleolar ankle fractures at 6 months after surgery.

	Operated Limb	Non-Operated Limb	*p*-Value	Operated Limb	Non-Operated Limb	*p*-Value
	(N = 14)	(N = 14)		(N = 21)	(N = 21)	
	Mean ± SD	Mean ± SD		Mean ± SD	Mean ± SD	
**Age**	45.6 ± 9.4			44.1 ± 11.1		
**Sex (% of women)**	50%			47.60%		
**Height (cm)**	166.9 ± 8.7			168.8 ± 9.2		
**Weight (kg)**	76.1 ± 9.5			77.9 ± 17.0		
**Immobilization time (weeks)**			3.4 ± 1.2			
**Unloading period (weeks)**			6.1 ± 1.3			
**Rehabilitation time (months)**			3.2 ± 2.5			
**AOFAS**	76.1 ± 9.5			84.4 ± 12.7		
**FDT_ROM_ (°)**	24.4 ± 8.7	29.0 ± 8.3	<0.001	30.4 ± 9.4	37.4 ± 6.1	<0.001
**ABD (%)**	28.2 ± 5.2	32.4 ± 8.2	0.025	30.4 ± 7.7	27.0 ± 7.2	<0.001

N: participants; SD: standard deviation; %: percentage; kg: kilograms; AOFAS: American Orthopedic Foot & Ankle Society hindfoot score; °: degrees; FDT_ROM_: ankle dorsiflexion range of motion; ABD: hip abductor strength normalized to body mass (%).

**Table 2:** Correlations between the muscle activity of the ankle and hip joint muscles of the operated limb at 6 months and 12 months after surgery during the Y-Balance Test (YBT), and YBT performance, dorsiflexion range of motion, abductor strength, and ankle functionality.

	Muscle Activity of the Ankle Muscles				Muscle Activity of the Hip Muscles			
	Operated Limb	Non-Operated Limb	Operated Limb	Non-Operated Limb	Operated Limb	Non-Operated Limb	Operated Limb	Non-Operated Limb
	6 Months		12 Months		6 Months		12 Months	
	*Anterior direction of the Y-Balance Test*							
YBT_A_-Distance	−0.185	−0.082	−0.281	−0.218	−0.365	0.058	−0.421	−0.013
FDT_ROM_ (°)	−0.603 ^GL^ ***	−0.371	−0.553	−0.253	−0.392	−0.279	−0.308	−0.136
ABD (%)	−0.340	−0.234	−0.427	−0.172	−0.153	−0.143	−0.355	0.023
AOFAS	−0.133	0.351	−0.410	−0.072	−0.047	0.062	−0.365	0.143
	*Posterior medial direction of the Y-Balance Test*							
YBT_PM_-Distance	−0.277	−0.259	−0.169	−0.207	−0.349	−0.124	−0.535	0.031
FDT_ROM_ (°)	−0.616 ^GL^ ***	−0.424	−0.699	−0.361	−0.586	−0.167	−0.371	0.054
ABD (%)	−0.330	−0.071	−0.344	−0.314	−0.33	−0.087	−0.553	0.206
AOFAS	−0.156	−0.098	−0.548	−0.413	−0.224	0.053	−0.452	0.283
	*Posterior lateral direction of the Y-Balance Test*							
YBT_PL_-Distance	0.104	−0.421	0.273	0.133	−0.388	0.269	−0.630	0.175
FDT_ROM_ (°)	−0.547	−0.235	−0.05	−0.164	−0.533	−0.125	−0.164	−0.179
ABD (%)	0.072	−0.279	0.235	−0.008	0.306	−0.072	−0.093	0.046
AOFAS	0.090	−0.374	0.029	−0.205	0.087	−0.160	−0.281	0.141

YBT: Y-Balance Test; A: anterior direction; PM: posteromedial direction; PL: posterolateral direction; FDT_ROM_: ankle dorsiflexion range of motion; ABD: hip abductor strength; (*p*-value from *r* Pearson, Bonferroni correction *p* < 0.004); *** (*p* < 0.001); GL: correlation between dorsal flexion and activation of the lateral gastrocnemius in the YBT_A_ and YBT_PM_ (−0. 734 ≤ r ≤ −0.782, *p* < 0.001).

## Data Availability

Data are contained within the article.

## Supplementary Materials

The following supporting information can be downloaded at: https://www.mdpi.com/article/10.3390/s24216798/s1: Table S1. Muscle activity of the 5 muscles in the operated and non-operated limb during stabilometry testing at 6 months after surgery; Table S2. Muscle activity of the 5 muscles in the operated and non-operated limbs during stabilometry testing at 12 months after surgery; Table S3. Muscle activity of the distal and proximal muscles of the affected and healthy legs during stabilometry at 6 months after surgery; Table S4. Muscle activity of the distal and proximal muscles of the affected and healthy legs during stabilometry at 12 months after surgery; Table S5. Muscle activity of the 5 muscles in the operated and non-operated limb during the Y-balance test at 6 months after surgery; Table S6. Muscle activity of the 5 muscles in the operated and non-operated limbs during the Y-Balance Test at 12 months after surgery; Table S7. Muscle activity of the distal and proximal muscles of the operated and non-operated limb during Y-Balance test at 6 months after surgery; Table S8. Muscle activity of the distal and proximal muscles of the operated and non-operated limbs during the Y-Balance Test at 12 months after surgery; Table S9. Correlations between stabilometry and ankle and hip joint muscle activity at 6 months and 12 months after surgery; Table S10a,b. Results of balance assessment at 6 months.
