# Supplementary material for: Changes in Motor Strategy and Neuromuscular Control During Balance Tasks in People with a Bimalleolar Ankle Fracture: A Preliminary and Exploratory Study
Source: Sensors (Basel). 2024 Oct 23;24(21):6798. doi: 10.3390/s24216798 (PMC11548516; doi:10.3390/s24216798)
Supplement: Supplementary file 1 [file sensors-24-06798-s001.zip › Table S1. Muscle activity of the 5 muscles in the operated and non-operated limb during stabilometry testing at 6 months after surgery.pdf]

Table S1. Muscle activity (% of the maximum voluntary contraction) of the 5 muscles in the operated and non-operated limbs during stabilometry testing at 6 months after surgery.

	Mean Amplitude				Coefficient of Variation			
	Operated limb Mean ± SD	Non-operated limb Mean ± SD	Effect size Hedges' g		Operated limb Mean ± SD	Non-operated limb Mean ± SD	Effect size Hedges' g	
**Unipodal test with eyes open**								
**Anterior tibialis**	10.8 ± 4.0	9.5 ± 4.9	0.28	(-0.26- 0.82)	71.4 ± 14.4*	86.4 ± 24.5	**-0.69**	**(-1.27- -0.08)**
**Peroneus longus**	23.7 ± 15.0	18.9 ± 7.9	0.24	(-0.30- 0.77)	76.3 ± 14.2	79.4 ± 18.3	-0.14	(-0.66- 0.40)
**Lateral gastrocnemius**	10.1 ± 4.6	10.0 ± 3.9	0.24	(-0.30- 0.77)	49.6 ± 8.2	44.8 ± 6.2	0.49	(-0.08- 1.04)
**Biceps femoris**	5.1 ± 3.8	3.9 ± 3.5	0.22	(-0.32- 0.74)	52.3 ± 16.0	51.2 ± 25.9	0.05	(-0.48- 0.57)
**Gluteus medius**	11.1 ± 8.0	12.1 ± 6.2	0.22	(-0.32- 0.74)	43.8 ± 11.0*	37.4 ± 14.6	**0.60**	**(0.01- 1.1**
**Unipodal test with eyes closed**								
**Anterior tibialis**	17.0 ± 5.0	17.0 ± 5.4	0.00	(-0.53- 0.53)	65.1 ± 13.8	69.3 ± 12.3	-0.28	(-0.83- 0.28)
**Peroneus longus**	33.0 ± 19.4	27.6 ± 10.4	0.20	(-0.33- 0.73)	70.9 ± 14.8	70.9 ± 16.4	0.00	(-0.55- 0.55)
**Lateral gastrocnemius**	13.1 ± 5.9	12.0 ± 3.9	0.22	(-0.32- 0.75)	54.7 ± 4.0	51.7 ± 11.8	0.13	(-0.42- 0.67)
**Biceps femoris**	8.2 ± 4.6	6.8 ± 5.7	0.21	(-0.33- 0.74)	57.3 ± 20.6	59.4 ± 21.0	-0.07	(-0.61- 0.48)
**Gluteus medius**	14.9 ± 10.8	14.8 ± 6.9	0.01	(-0.52- 0.53)	58.0 ± 17.9	55.0 ± 18.4	0.12	(-0.43- 0.67)
**Tandem test**								
**Anterior tibialis**	6.6 ± 5.2	7.8 ± 6.2	-0.17	(-0.70- 0.36)	94.4 ± 37.0	90.4 ± 35.0	0.08	(-0.45- 0.60)
**Peroneus longus**	11.8 ± 7.9	10.9 ± 6.9	0.12	(-0.41- 0.65)	80.7 ± 31.6	85.4 ± 26.4	-0.11	(-0.64- 0.41)
**Lateral gastrocnemius**	6.5 ± 4.2	6.6 ± 4.5	-0.02	(-0.55- 0.50)	59.6 ± 20.2	55.9 ± 24.5	0.17	(-0.36- 0.69)
**Biceps femoris**	2.9 ± 3.3	2.5 ± 2.9	0.11	(-0.41- 0.64)	46.4 ± 23.4	49.6 ± 38.9	-0.11	(-0.63- 0.42)
**Gluteus medius**	7.6 ± 4.3	7.9 ± 3.7	-0.04	(-0.56- 0.49)	47.8 ± 42.3	45.0 ± 29.2	0.07	(-0.44- 0.58)

SD: standard deviation; * differences between operated and non-operated limbs in each stabilometry test, $p<0.05$ with Bonferroni adjustment; effect sizes were calculated using the Hedges' g index and are presented as mean (95% confidence interval).
