# Supplementary material for: Changes in Motor Strategy and Neuromuscular Control During Balance Tasks in People with a Bimalleolar Ankle Fracture: A Preliminary and Exploratory Study
Source: Sensors (Basel). 2024 Oct 23;24(21):6798. doi: 10.3390/s24216798 (PMC11548516; doi:10.3390/s24216798)
Supplement: Supplementary file 1 [file sensors-24-06798-s001.zip › Table S10a,b. Results of Balance assesment att 6 months.pdf]

Table S10a. Static balance at the 6-month assessment after surgery in 14 patients

Unipodal open eyes	Operated limb<br>Mean ± SD	Non-operated limb<br>Mean ± SD	*Effect size*<br>*Hedges' g*
COP distance (mm)	613.8 ± 200.6	584.1 ± 216.1	0.13 (-0.28; 0.56)
V(mm/sg)	33.0 ± 4.3	20.1 ± 6.4	0.30 (-0.39; 1.12)
LSF	1.6 ± 0.5	1.5 ± 0.7	0.15 (-0.54; 0.86)
**Unipodal closed eyes**			
COP distance (mm)	1157.6 ± 411.6	1293.6 ± 315.3	-0.33 (-01.10; 0.38)
V (mm/sg)	37.6 ± 1.5	43.1 ± 1.1	-3.78 (-6.20; -2.14)
LSF	0.9 ± 0.2	0.7 ± 0.2	**0.91 (0.14; 1.84) ***
**Tandem**			
COP distance (mm)	665.7 ± 328.7	541.9 ± 342.3	0.35 (-0.14; 0.86)
V (mm/sg)	34.5 ± 3.3	18.1 ± 1.1	0.61 (-0.17; 1.46)
LSF	1.1 ± 0.5	1.0 ± 0.5	0.19 (-0.51; 0.90)

SD: standard deviation; COP distance: distance covered by the center of pressure; VCOP: mean velocity of the center of pressure; LSF: length/surface; the comparison is statistically significant when the confidence interval of the effect size does not cross the zero value.

Table S10b. Dynamic balance at the 6-month assessment after surgery in 14 patients

YBT Direction	Operated limb<br>Mean ± SD	Non-operated limb<br>Mean ± SD	*Effect size*<br>*Hedges' g*
Anterior	62.2 ± 11.8	69.9 ± 10.4	**-0.64 (-1.19; -0.18)**
Posteromedial	102.7 ± 10.9	108.0 ± 10.8	**-0.45 (-0.77; -0.19)**
Posterolateral	98.4 ± 14.9	102.2 ± 13.5	-0.25 (-0.70; 0.18)

SD: standard deviation; YBT: Y-Balance Test; the *comparison is statistically significant when the confidence interval of the effect size does not cross the zero value.*
