# Supplementary material for: Changes in Motor Strategy and Neuromuscular Control During Balance Tasks in People with a Bimalleolar Ankle Fracture: A Preliminary and Exploratory Study
Source: Sensors (Basel). 2024 Oct 23;24(21):6798. doi: 10.3390/s24216798 (PMC11548516; doi:10.3390/s24216798)
Supplement: Supplementary file 1 [file sensors-24-06798-s001.zip › Table S2. Muscle activity of the 5 muscles in the operated and non-operated limb during stabilometry testing at 12 months after surgery.pdf]

Table S2. Muscle activity (% of the maximum voluntary contraction) of the 5 muscles in the operated and non-operated limbs during stabilometry testing at 12 months after surgery.

**Unipodal test with eyes open**						
	**Mean Amplitude**			**Coefficient of Variation**		
	**Operated limb**	**Non-operated limb**	**Effect size**	**Operated limb**	**Non-operated limb**	**Effect size**
	**Mean ± SD**	**Mean ± SD**	**Hedges' g**	**Mean ± SD**	**Mean ± SD**	**Hedges' g**
**Anterior tibialis**	8.9 ± 5.7	7.2 ± 5.0	0.34 (-0.10- 0.78)	52.8 ± 37.0	51.9 ± 32.6	0.02 (-0.41- 0.45)
**Peroneus longus**	16.9 ± 15.0	13.6 ± 8.9	0.23 (-0.21- 0.66)	50.4 ± 28.9	48.8 ± 32.6	0.04 (-0.39- 0.47)
**Lateral gastrocnemius**	7.1 ± 6.2	9.4 ± 8.6	-0.30 (-0.74- 0.14)	54.2 ± 52.8	43.3 ± 37.2	0.16 (-0.28- 0.59)
**Biceps femoris**	5.6 ± 4.1	6.9 ± 4.5	-0.27 (-0.70- 0.17)	38.9 ± 17.3	33.5 ± 14.2	0.37 (-0.08- 0.81)
**Gluteus medius**	11.7 ± 11.1	13.7 ± 18.9	-0.10 (-0.53- 0.33)	38.7 ± 17.1	37.9 ± 17.0	0.03 (-0.40- 0.46)
**Unipodal test with eyes closed**						
**Anterior tibialis**	18.3 ± 5.4	17.7 ± 5.8	0.10 (-0.36- 0.56)	68.5 ± 18.5	69.6 ± 13.6	-0.08 (-0.53- 0.37)
**Peroneus longus**	32.6 ± 9.8*	24.3 ± 6.9	0.87 (-0.31- 1.40)	66.1 ± 16.4*	77.0 ± 18.3	-0.51 (-0.98- -0.02)
**Lateral gastrocnemius**	17.2 ± 9.8	14.8 ± 9.9	0.20 (-0.27- 0.66)	51.5 ± 16.0	51.9 ± 15.0	-0.02 (-0.48- 0.43)
**Biceps femoris**	8.5 ± 4.8	7.8 ± 7.0	0.10 (-0.36- 0.56)	60.4 ± 25.1	59.1 ± 25.9	0.04 (-0.42- 0.49)
**Gluteus medius**	20.9 ± 15.0	14.9 ± 11.9	0.48 (-0.02- 0.96)	49.5 ± 25.6	47.0 ± 18.8	0.09 (-0.36- 0.54)
**Tandem test**						
**Anterior tibialis**	6.4 ± 3.3	4.9 ± 3.4	0.40 (-0.08- 0.86)	77.7 ± 35.2	80.0 ± 31.7	-0.06 (-0.50- 0.38)
**Peroneus longus**	13.8 ± 8.1	11.0 ± 6.1	0.36 (-0.11- 0.83)	71.6 ± 34.6	81.3 ± 27.5	-0.25 (-0.69- 0.21)
**Lateral gastrocnemius**	5.9 ± 3.3*	8.5 ± 4.5	**-0.63 (-1.13- -0.13)**	43.5 ± 23.9	46.0 ± 24.7	-0.07 (-0.51- 0.37)
**Biceps femoris**	2.8 ± 1.8	4.0 ± 5.0	-0.25 (-0.70- 0.21)	32.0 ± 21.2	42.0 ± 33.5	-0.33 (-0.77- 0.13)
**Gluteus medius**	9.1 ± 6.2	7.2 ± 5.1	0.37 (-0.10- 0.84)	41.7 ± 52.5	33.9 ± 15.6	0.14 (-0.30- 0.58)

SD: standard deviation; * differences between operated vs. non-operated limbs in each stabilometry test, $p<0.05$ with Bonferroni adjust; effect sizes were calculated using the Hedges' g index and are presented as mean (95% confidence interval).
