# Supplementary material for: Changes in Motor Strategy and Neuromuscular Control During Balance Tasks in People with a Bimalleolar Ankle Fracture: A Preliminary and Exploratory Study
Source: Sensors (Basel). 2024 Oct 23;24(21):6798. doi: 10.3390/s24216798 (PMC11548516; doi:10.3390/s24216798)
Supplement: Supplementary file 1 [file sensors-24-06798-s001.zip › Table S3. Muscle activity of the distal and proximal muscles of the affected and healthy leg during stabilometry at 6 months after surgery..pdf]

Table S3. Muscle activity of the distal and proximal muscles of the affected and healthy legs during stabilometry at 6 months after surgery.

**Unipodal open eyes**		**Operated limb Mean ± SD**	**Non-operated limb Mean ± SD**	***Limb F(p)***	***Effect size***
Mean of activation	Ankle	14.9 ± 6.0*	12.8 ± 4.6*	0.849 (0.375)	0.37 (-0.35; 1.12)
	Hip	8.1 ± 4.7*	7.9 ± 3.8*		0.03 (0.50; 0.56)
	Joint F(p)	20.753 (<0.001)		*Interaction*	
	Effect size	**-1.14 (-2.13; -0.32)**	**1.06 (0.41; 1.83)**	1.006 (0.336)	
Coefficient of variation	Ankle	65.8 ± 9.6*	70.2 ± 13.4*	0.016 (0.900)	-0.35 (-1.04; 0.30)
	Hip	48.1 ± 11.2*	44.3 ± 17.4*		0.22 (-0.21; 0.72)
	Joint F(p)	26.756 (<0.001)		*Interaction*	
	Effect size	**1.86 (0.89; 3.04)**	**1.56 (0.76; 2.55)**	3.115 (0.103)	
**Unipodal closed eyes**		**Operated limb**	**Non-operated limb**	***Limb***	***Effect size***
Mean of activation	Ankle	21.0 ± 7.9*	18.9 ± 4.9*	0.675 (0.427)	0.30 (-0.49; 1.12)
	Hip	11.6 ± 5.8*	11.5 ± 1.6*		0.14 (-0.42; 0.70)
	Joint F(p)	17.005 (0.001)			
	Effect size	**1.27 (0.14; 2.53)**	**1.52 (0.75; 2.46)**	0.675 (0.427)	
Coefficient of variation	Ankle	63.6 ± 12.9	64.0 ± 10.3	0.46)000 (0.997)	-0.03 (-0.77; 0.71)
	Hip	57.6 ± 17.2	57.2 ± 15.6		-0.31 (-1.22; 0.57)
	Joint F(p)	2.830 (0.121)		*Interaction*	
	Effect size	0.36 (-0.23; 1.00)	0.49 (-0.48; 1.49)	0.010 (0.923)	
**Tandem**		**Operated limb**	**Non-operated limb**	***Limb***	***Effect size***
Mean of activation	Ankle	8.3 ± 4.8*	8.4 ± 5.3*	0.000 (0.998)	-0.02 (-0.46; 0.42)
	Hip	5.3 ± 3.4*	5.2 ± 2.2*		0.03 (-0.75; 0.82)
	Joint F(p)	9.248 (0.009)		*Interaction*	
	Effect size	**0.65 (0.15; 1.27)**	**0.65 (0.11; 1.44)**	0.061 (0.809)	
Coefficient of variation	Ankle	78.2 ± 18.1*	77.2 ± 21.6*	0.006 (0.941)	0.05 (-0.58; 0.67)
	Hip	47.1 ± 25.3*	47.3 ± 27.6*		-0.01 (-0.53; 0.51)
	Joint F(p)	33.585 (<0.001)		*Interaction*	
	Effect size	**1.31 (0.60; 2.20)**	**1.21 (0.47; 1.91)**	0.019 (0.892)	

*Two-way repeated measures ANOVAs, with limb (operated vs. non-operated) and joint (ankle and hip) being the within-group factors. The main effects of the ANOVAs (leg and joint) and interactions are presented as F score (p); * p<0.05 with Bonferroni correction. Descriptive data are presented as mean and standard deviation (SD). Effect sizes were calculated using the Hedges' g index and are presented as mean (95% confidence interval).*
