# Supplementary material for: Changes in Motor Strategy and Neuromuscular Control During Balance Tasks in People with a Bimalleolar Ankle Fracture: A Preliminary and Exploratory Study
Source: Sensors (Basel). 2024 Oct 23;24(21):6798. doi: 10.3390/s24216798 (PMC11548516; doi:10.3390/s24216798)
Supplement: Supplementary file 1 [file sensors-24-06798-s001.zip › Table S4. Muscle activity of the distal and proximal muscles of the affected and healthy leg during stabilometry at 12 months after surgery..pdf]

Table S4. Muscle activity of the distal and proximal muscles of the affected and healthy legs during stabilometry at 12 months after surgery.

**Unipodal open eyes**		**Operated limb**	**Non-operated limb**	***Limb***	
		**Mean ± SD**	**Mean ± SD**	***F(p)***	***Effect size***
Mean of activation	Ankle	11.0 ± 8.0	11.1 ± 6.4	0.050 (0.826)	0.11 (-0.23; 0.48)
	Hip	8.7 ± 6.1	10.3 ± 10.3		-0.18 (-0.75; 0.37)
	Joint F(p)	0.585 (0.454)			*Interaction*
	Effect size	0.30 (-0.06; 0.70)	-0.02 (-0.48; 0.42)		1.576 (0.224)
Coefficient of variation	Ankle	52.5 ± 27.0	48.0 ± 29.1	0.620 (0.441)	0.15 (-0.47; 0.79)
	Hip	38.8 ± 12.8	35.7 ± 11.3		0.24 (-0.35; 0.85)
	Joint F(p)	9.150 (0.007)			*Interaction*
	Effect size	0.62 (-0.00; 1.28)	0.51 (-0.05; 1.16)		0.019 (0.892)
**Unipodal closed eyes**		**Operated limb**	**Non-operated limb**	**Limb**	***Effect size***
Mean of activation	Ankle	22.7 ± 6.3*,*	18.9 ± 5.2*·*	0.803 (0.388)	**0.62 (0.20; 1.10)**
	Hip	14.8 ± 8.3*	11.4 ± 7.9*		0.40 (-0.04; 0.87)
	Joint F(p)	26.282 (>0.001)			*Interaction*
	Effect size	**1.02 (0.37; 1.75)**	**0.99 (0.55; 1.67)**		0.257 (0.621)
Coefficient of variation	Ankle	62.0 ± 12.5	66.2 ± 13.4*	0.107 (0.748)	-0.30 (-0.71; 0.09)
	Hip	54.9 ± 22.0	53.0 ± 19.7*		0.09 (-0.47; 0.65)
	Joint F(p)	8.129 (0.011)			*Interaction*
	Effect size	0.38 (-0.22; 1.01)	**0.74 (0.15; 1.39)**		0.627 (0.439)
**Tandem**		**Operated limb**	**Non-operated limb**	**Limb**	***Effect size***
Mean of activation	Ankle	9.6 ± 5.0	8.2 ± 3.3*	0.000 (0.998)	0.10 (-0.18; 0.40)
	Hip	7.5 ± 8.1	5.6 ± 4.7*		0.10 (-0.56; 0.74)
	Joint F(p)	9.248 (0.009)			*Interaction*
	Effect size	0.26 (-0.05; 0.68)	**0.60 (0.10; 1.19)**		0.06 (0.809)
Coefficient of variation	Ankle	64.3 ± 26.0*	69.0 ± 22.3*	0.245 (0.626)	-0.19 (-0.76; 0.37)
	Hip	36.8 ± 33.9*	38.1 ± 21.6*		-0.04 (-0.62; 0.53)
	Joint F(p)	42.629 (>0.001)			*Interaction*
	Effect size	**0.87 (0.31; 1.51)**	**1.35(0.78; 0.02)**		0.135 (0.718)

*Two-way repeated measures ANOVAs, with limb (operated vs. non-operated) and joint (ankle and hip) being the within-group factors. The main effects of the ANOVAs (leg and joint) and interactions are presented as F score (p); * p<0.05 with Bonferroni correction. Descriptive data are presented as mean and standard deviation (SD). Effect sizes were calculated using the Hedges' g index and are presented as mean (95% confidence interval).*
