# Supplementary material for: Changes in Motor Strategy and Neuromuscular Control During Balance Tasks in People with a Bimalleolar Ankle Fracture: A Preliminary and Exploratory Study
Source: Sensors (Basel). 2024 Oct 23;24(21):6798. doi: 10.3390/s24216798 (PMC11548516; doi:10.3390/s24216798)
Supplement: Supplementary file 1 [file sensors-24-06798-s001.zip › Table S5 . Muscle activity of the 5 muscles in the operated and non-operated limb during the Y-balance test at 6 months after surgery..pdf]

Table S5 . Muscle activity (% of maximal voluntary contraction) of the 5 muscles in the affected and healthy leg during the Y-balance test at 6 months after surgery.

	YBT_A				YBT_{PM}				YBT_{PL}			
**Muscle**	**Operated Limb**	**Non-operated limb**	**Effect Size**		**)perated Lim**	**Non-operated limb**	**Effect Size**		**Operated Limb**	**Non-operated limb**	**Effect Size**	
	**Mean ± SD**	**Mean ± SD**	**Hedges' g**		**Mean ± SD**	**Mean ± SD**	**Hedges' g**		**Mean ± SD**	**Mean ± SD**	**Hedges' g**	
**Anterior tibialis**	**23.9 ± 11.9** *	**32.4 ± 10.1**	**-0.61**	**(-1.16: -0.04)**	31.3 ± 14.4	41.6 ± 13.1	-0.50	(-1.05: 0.07)	30.9 ± 10.0	39.7 ± 14.3	-0.57	(-1.15: 0.04)
**Peroneus longus**	32.8 ± 15.8	41.3 ± 12.3	-0.37	(-0.89: 0.17)	34.0 ± 15.4	39.4 ± 12.4	-0.22	(-0.75: 0.32)	38.1 ± 17.7	43.6 ± 12.4	-0.24	(-0.79: 0.32)
**Lateral gastrocnemius**	28.0 ± 12.5	28.7 ± 15.4	-0.04	(-0.62: 0.53)	19.5 ± 10.0	16.5 ± 10.4	0.54	(-0.04: 1.09)	16.3 ± 8.7	17.2 ± 16.2	0.49	(-0.10: 1.06)
**Biceps femoris**	21.4 ± 9.6	15.3 ± 12.1	0.47	(-0.08: 1.00)	17.6 ± 7.5	16.9 ± 15.2	0.07	(-0.46: 0.60)	20.3 ± 13.7	22.8 ± 20.3	-0.17	(-0.71: 0.39)
**Gluteus medius**	33.3 ± 17.4	26.3 ± 11.7	0.32	(-0.21: 0.83)	**41.6 ± 15.9** *	**29.9 ± 16.6**	**0.70**	(0.10: 1.29)	27.4 ± 16.3	31.0 ± 19.1	-0.12	(-0.66: 0.44)

YBTA: Y balance test anterior direction; YBTPM: Y balance test posteromedial direction; YBTPL: Y balance test posterolateral direction; SD: standard deviation; * differences between affected vs healthy leg in each direction of the Y-balance test; $p<0.05$ with Bonferroni adjust.
