# Supplementary material for: Changes in Motor Strategy and Neuromuscular Control During Balance Tasks in People with a Bimalleolar Ankle Fracture: A Preliminary and Exploratory Study
Source: Sensors (Basel). 2024 Oct 23;24(21):6798. doi: 10.3390/s24216798 (PMC11548516; doi:10.3390/s24216798)
Supplement: Supplementary file 1 [file sensors-24-06798-s001.zip › Table S6 . Muscle activity of the 5 muscles in the operated and non-operated limb during the Y-balance test at 12 months after surgery..pdf]

Table S6 . Muscle activity (% of maximal voluntary contraction) of the 5 muscles in the affected and healthy leg during the Y-balance test at 12 months after surgery.

	YBT_A				YBT_{PM}				YBT_{PL}			
**Muscle**	**Operated Limb**	**Non-operated limb**	**Effect Size**		**Operated Limb**	**Non-operated limb**	**Effect Size**		**Operated Limb**	**Non-operated limb**	**Effect Size**	
	**Mean ± SD**	**Mean ± SD**	**Hedges' g**		**Mean ± SD**	**Mean ± SD**	**Hedges' g**		**Mean ± SD**	**Mean ± SD**	**Hedges' g**	
**Anterior tibialis**	28.9 ± 10.8	33.8 ± 14.6	-0.30	(-0.74- 0.14)	34.6 ± 11.8	40.8 ± 15.7	-0.35	(-0.80- 0.11)	31.0 ± 21.8	35.3 ± 19.6	-0.14	(-0.59- 0.30)
**Peroneus longus**	38.0 ±12.5	36.1 ± 13.8	0.09	(-0.34- 0.52)	43.2 ± 19.0	38.0 ± 11.1	0.24	(-0.21- 0.68)	37.4 ± 25.0	32.3 ± 15.9	0.29	(-0.17- 0.73)
**Lateral gastrocnemius**	27.4 ±15.8	28.1 ± 17.1	-0.04	(-0.47- 0.39)	18.3 ± 9.9	19.4 ± 13.0	-0.08	(-0.52- 0.36)	14.0 ± 9.0	15.5 ± 9.0	-0.12	(-0.56- 0.32)
**Biceps femoris**	21.0 ±13.2	15.2 ± 9.6	0.46	(0.00- 0.91)	16.6 ± 8.1	17.4 ± 11.0	-0.08	(-0.52- 0.36)	15.5 ± 13.1	14.20± 11.0	0.09	(-0.35- 0.53)
**Gluteus medius**	29.5 ±17.1	27.9 ± 17.7	0.07	(-0.36- 0.50)	33.3 ± 18.3	32.6 ± 11.9	0.03	(-0.41- 0.47)	27.3 ± 21.0	32.5 ± 16.3	-0.16	(-0.60- 0.29)

YBT_A: Y balance test dirección anterior; YBT_{PM}: Y balance test dirección posteromedial; YBT_{PL}: Y balance test dirección posterolateral; SD: desviación estándar; se marcan mediante números las diferencias entre los músculos en cada dirección del Y-balance test; se marcan mediante letras las diferencias de cada grupo muscular entre las direcciones del Y-balance test; * diferencias entre pierna afecta vs sana en cada dirección del Y-balance test; $p< 0.05$.
