# Supplementary material for: Changes in Motor Strategy and Neuromuscular Control During Balance Tasks in People with a Bimalleolar Ankle Fracture: A Preliminary and Exploratory Study
Source: Sensors (Basel). 2024 Oct 23;24(21):6798. doi: 10.3390/s24216798 (PMC11548516; doi:10.3390/s24216798)
Supplement: Supplementary file 1 [file sensors-24-06798-s001.zip › Table S7. Muscle activity of the distal and proximal muscles of the operated and non-operated limb during Y-Balance test at 6 months after surgery..pdf]

Table S7. Muscle activity of the distal and proximal muscles of the operated and non-operated limbs during Y-Balance Test at 6 months after surgery.

YBT Direction		Operated limb<br>Mean ± SD	Non-operated limb<br>Mean ± SD	***Limb***<br>*F(p)*	***Effect size***
**Anterior**	Distal	Distal	34.1 ± 7.8*	0.016 (0.901)	-0.62 (-1.33: 0.03)
	Proximal	Proximal	20.8 ± 9.6*,*		**0.62 (0.04; 1.26)**
	Joint F(p)	Joint F(p)		*Interaction*	
	Effect size	Effect size	**1.42 (0.68; 2.33)**	30.806 (>0.001)	
**Posteromedial**	Distal	Distal	31.1 ± 6.7*	0.777 (0.398)	-0.33 (-0.97; 0.27)
	Proximal	Proximal	22.4 ± 13.5*		0.55 (-0.15; 1.30)
	Joint F(p)	Joint F(p)		*Interaction*	
	Effect size	Effect size	**0.71 (0.09; 1.51)**	4.121 (0.065)	
**Posterolateral**	Distal	28.4 ± 8.7	31.0 ± 8.5	2.319 (0.156)	-0.28 (-0.82; 0.23)
	Proximal	23.8 ± 11.5	26.5 ± 15.7		-0.36 (-0.89; 0.08)
	Joint F(p)	4.742 (0.052)		*Interaction*	
	Effect size	0.40 (-0.04; 0.92)	0.30 (-0.22; 0.92)	0.000 (0984)	

*YBT: Y-Balance Test. Two-way repeated measures ANOVAs, with limb (operated vs. non-operated) and joint (ankle and hip) being the within-group factors. The main effects of the ANOVAs (limb and joint) and interactions are presented as F score (p); * p<0.05 with Bonferroni correction. Descriptive data are presented as mean and standard deviation (SD). Effect sizes were calculated using the Hedges' g index and are presented as mean (95% confidence interval).*
