# Supplementary material for: Changes in Motor Strategy and Neuromuscular Control During Balance Tasks in People with a Bimalleolar Ankle Fracture: A Preliminary and Exploratory Study
Source: Sensors (Basel). 2024 Oct 23;24(21):6798. doi: 10.3390/s24216798 (PMC11548516; doi:10.3390/s24216798)
Supplement: Supplementary file 1 [file sensors-24-06798-s001.zip › Table S8. Muscle activity of the distal and proximal muscles of the operated and non-operated limb during Y-Balance test at 12 months after surgery..pdf]

Table S8. Muscle activity of the distal and proximal muscles of the operated and non-operated limbs during Y-Balance Test at 12 months after surgery.

**YBT Direction**		**Operated limb Mean ± SD**	**Non-operated limb Mean ± SD**	***Limb*** *F(p)*	***Effect size***
**Anterior**	Distal	31.4 ± 6.9 +	32.2 ± 7.3 +	0.418 (0.526)	-0.17 (-0.57; 0.21)
	Proximal	25.2 ± 11.3+	21.4 ± 11.7 +		0.32 (-0.19; 0.85)
	Joint F(p)	16.623 (0.001)		*Interaction*	
	Effect size	**0.62 (0.10; 1.22)**	**1.03 (0.48; 1.71)**	3.245 (0.088)	
		32.0 ± 8.7 +	32.4 ± 7.3 +	0.011 (0.917)	-0.05 (-0.47; 0.38)
**Posteromedial**	Distal	25.0 ± 11.1 +	25.0 ± 8.5 +		0 (-0.55; 0.55)
	Proximal	15.917 (0.001)		*Interaction*	
	Joint F(p)	**0.67 0.14; 1.25)**	**0.90 (0.34; 1.51)**	0.022 (0.884)	
	Effect size	27.5 ± 15.2	27.0 ± 9.4	0.040 (0.844)	0 (-0.43; 0.43)
		21.4 ± 14.6	23.1 10.8		-0.13 (-0.77; 0.51)
**Posterolateral**	Distal	4.580 (0.046)		*Interaction*	
	Proximal	0.39 (-0.01; 0.81)	0.39 (-0.13; 0.89)	0.441 (0.515)	

*YBT: Y-Balance Test. Two-way repeated measures ANOVAs, with limb (operated vs. non-operated) and joint (ankle and hip) being the within-group factors. The main effects of the ANOVAs (limb and joint) and interactions are presented as F score (p); * p<0.05 with Bonferroni correction. Descriptive data are presented as mean and standard deviation (SD). Effect sizes were calculated using the Hedges' g index and are presented as mean (95% confidence interval).*
