# Supplementary material for: Changes in Motor Strategy and Neuromuscular Control During Balance Tasks in People with a Bimalleolar Ankle Fracture: A Preliminary and Exploratory Study
Source: Sensors (Basel). 2024 Oct 23;24(21):6798. doi: 10.3390/s24216798 (PMC11548516; doi:10.3390/s24216798)
Supplement: Supplementary file 1 [file sensors-24-06798-s001.zip › Table S9. Correlations between stabilometry and ankle and hip joint muscle activity at 6 months and 12 months after surgery..pdf]

Table S9. Correlations between stabilometry and ankle and hip joint muscle activity at 6 months and 12 months after surgery.

			DIS_{COP}		V_{COP}		LFS	
			6 months	12 months	6 months	12 months	6 months	12 months
**Unipodal eyes open**	Mean	Ankle	-0.229	0.108	0.210	0.109	-0.618*	0.350
		Hip	0.211	0.222	0.357	0.223	-0.164	0.196
	Coefficient variation	Ankle	0.092	0.141	0.111	0.141	0.291	0.019
		Hip	0.152	-0.096	0.006	-0.096	-0.401	0.018
**Unipodal eyes closed**	Mean	Ankle	-0.453	-0.166	-0.367	-0.148	0.317	0.160
		Hip	0.526	0.090	0.542	0.007	0.480	0.324
	Coefficient variation	Ankle	0.499	0.681	0.485	0.617	-0.182	-0.292
		Hip	0.345	0.271	0.347	0.319	-0.666*	-0.051
**Tandem**	Mean	Ankle	0.563*	0.314	-0.020	0.315	-0.589*	0.272
		Hip	0.447	0.220	0.069	0.221	0.452	-0.010
	Coefficient variation	Ankle	-0.096	0.087	0.272	0.088	0.207	-0.032
		Hip	0.429	0.109	0.113	0.109	-0.410	-0.106

COP distance: distance covered by the center of pressure; VCOP: mean velocity of the center of pressure; LSF: length/surface.
